# HuR silencing elicits oxidative stress and DNA damage and sensitizes human triple-negative breast cancer cells to radiotherapy

**DOI:** 10.18632/oncotarget.11706

**Published:** 2016-08-30

**Authors:** Meghna Mehta, Kanthesh Basalingappa, James N. Griffith, Daniel Andrade, Anish Babu, Narsireddy Amreddy, Ranganayaki Muralidharan, Myriam Gorospe, Terence Herman, Wei-Qun Ding, Rajagopal Ramesh, Anupama Munshi

**Affiliations:** ^1^ Department of Radiation Oncology, The University of Oklahoma Health Sciences Center, Oklahoma City, Oklahoma, USA; ^2^ Department of Pathology, The University of Oklahoma Health Sciences Center, Oklahoma City, Oklahoma, USA; ^3^ Stephenson Cancer Center, The University of Oklahoma Health Sciences Center, Oklahoma City, Oklahoma, USA; ^4^ National Institute on Aging, National Institutes of Health, Baltimore, Maryland, USA; ^5^ Graduate Program in Biomedical Sciences, The University of Oklahoma Health Sciences Center, Oklahoma City, Oklahoma, USA

**Keywords:** HuR, breast cancer, DNA repair, radiation, siRNA

## Abstract

HuR is an mRNA-binding protein whose overexpression in cancer cells has been associated with poor prognosis and resistance to therapy. While reports on HuR overexpression contributing to chemoresistance exist, limited information is available on HuR and radioresistance especially in triple-negative breast cancer (TNBC).

In this study we investigated the role of HuR in radiation resistance in three TNBC (MDA-MB-231, MDA-MB-468 and Hs578t) cell lines. Endogenous HuR expression was higher in TNBC cells compared to normal cells. siRNA mediated knockdown of HuR (siHuR) markedly reduced HuR mRNA and protein levels compared to scrambled siRNA (siScr) treatment. Further, siHuR treatment sensitized TNBC cells to ionizing radiation at 2 Gy compared to siScr treatment as evidenced by the significant reduction in clonogenic cell survival from 59%, 49%, and 65% in siScr-treated cells to 40%, 33%, and 46% in siHuR-treated MDA-MB-231, MDA-MB-468 and Hs578t cells, respectively. Molecular studies showed increased ROS production and inhibition of thioredoxin reductase (TrxR) in HuR knockdown cells contributed to radiosensitization. Associated with increased ROS production was evidence of increased DNA damage, demonstrated by a significant increase (*p* < 0.05) in γ-H2AX foci that persisted for up to 24 h in siHuR plus radiation treated cells compared to control cells. Further, comet assay revealed that HuR-silenced cells had larger and longer-lasting tails than control cells, indicating higher levels of DNA damage. In conclusion, our studies demonstrate that HuR knockdown in TNBC cells elicits oxidative stress and DNA damage resulting in radiosensitization.

## INTRODUCTION

Triple negative breast cancer (TNBC) is a distinct subset of breast cancer characterized by aggressive clinical behavior with limited treatment options and very poor prognosis. Increased locoregional recurrence (LRR) is a feature of TNBC and the frequent development of resistance to current standard therapies results in disease recurrence and metastasis for which there are few effective treatments [1, 2]. While radiation therapy is an integral component of treatment to gain local control and palliate distant metastasis, development of resistance to therapy due to alterations in molecular pathways limits the success of radiotherapy. Therefore, unraveling the underlying mechanism controlling the development of resistance to radiation therapy can help delay or eliminate the development of resistance and pave the way for designing effective radiosensitizers.

Ionizing radiation causes both direct and indirect damage to cells. The major cellular target of ionizing radiation is DNA which can be damaged directly by ionizing radiation, resulting in DNA double-strand breaks (DSBs), or indirectly through generation of reactive oxygen species (ROS) [3-5]. Exposure to clinically relevant doses of ionizing radiation elicits genotoxic stress by triggering increased levels of ROS which creates oxidative stress and disturbs the redox balance within the cells leading to generation of DNA damage [6, 7]. In order to effectively eliminate ROS and to cope with the damaging effects from ROS mediated stress, tumor cells have developed antioxidant defense mechanisms which include non-enzymatic radical scavengers and cellular antioxidant systems, of which the thioredoxin (Trx) system is a key player [8, 9]. The thioredoxin system is an oxidative stress response system and includes thioredoxin (Trx), thioredoxin reductase (TrxR), and thioredoxin interacting protein (TxNIP) [10]. Thioredoxin reductase (TrxR) is reported to be overexpressed in many aggressive cancers and plays a crucial role in redox balance and antioxidant function, including defense of oxidative stress [11,12]. Inhibition of the thioredoxin (Trx) system can disrupt the homeostasis of cancer cells causing a dramatic imbalance between the formation and the removal of ROS. Therefore, agents that can inhibit TrxR have the potential to be developed as novel anticancer agents and radiosensitizers [13, 14].

Altered gene expression is a critical point of dysregulation in cancer and increasing evidence suggests that post-transcriptional processing of mRNA transcripts plays a major role in ensuring proper gene expression [15]. These post-transcriptional events are mediated by a myriad of RNA-binding proteins (RBPs), which are master regulators of mRNA processing and translation and are often aberrantly expressed in cancer. Human antigen R (HuR), is an RNA binding protein (RBP) that is a member of the embryonic lethal abnormal vision (ELAV) family comprising of four vertebrate members - HuB, HuC and HuD which are primarily neuronal proteins, and HuR which is widely expressed in all proliferating cells [16, 17]. HuR has a variety of biological functions which are all based on its ability to bind to short U-rich or AU-rich ARE sequence motifs in the 3′-untranslated regions (UTRs) of target mRNAs regulating their splicing, export, stability, and translation [18-21]. HuR has been demonstrated to control the expression of genes involved in cell cycle and apoptosis, metastasis, angiogenesis and hypoxia [22-28]. Consequently, HuR has been proposed to play a pivotal role in tumor formation, growth, and metastasis [29-31]. Given its central regulatory role in diverse cellular processes, the involvement of deregulated HuR in carcinogenesis through aberrant orchestration of cellular responses is increasingly apparent. HuR function appears to be closely linked to its subcellular localization [21, 32]. Although HuR is primarily located in the nucleus, its biological function is mainly executed by its translocation to the cytoplasm which can be modulated by numerous stimuli, both endogenous and external such as exposure to ultraviolet radiation, oxidative stress, DNA damage, and heat shock [17, 32-35]It has been demonstrated that increased cytoplasmic HuR expression is a poor prognostic factor and is associated with aggressiveness in several types of carcinoma, including those of the lung, colon, esophagus, ovary and breast [36-40].

However, the interactions between HuR effects and chemotherapy induced cytotoxic effects are complex. Increase in HuR was found to correlate with improved response to treatment with adjuvant gemcitabine in pancreatic cancer; in contrast, HuR expression was shown to play a role in paclitaxel resistance in ovarian cancer [41-43]. In breast cancer cells, the increased presence of HuR in the cytoplasm has been associated with increased doxorubicin-induced apoptosis, but also with the development of tamoxifen resistance [44, 45]. Taken together, these studies implicate HuR in regulating drug killing in various cancers. While HuR has been directly linked to chemotherapy response, a possible role for HuR in influencing radiotherapy response has not been addressed previously.

In the present study we investigated whether silencing HuR sensitized TNBC cells to ionizing radiation. We demonstrate that silencing HuR resulted in radiosensitization of TNBC cells through enhanced ROS accumulation along with an inhibition of the thioredoxin reductase (TrxR) system. Further, silencing HuR also significantly delayed radiation induced DNA double strand breaks (DNA DSBs) as evident by prolonged expression of γ-H2AX foci. Results from our studies suggest HuR targeted therapy in context with radiation could be effective against TNBCs.

## RESULTS

### HuR expression is higher in human breast cancer cell lines

**Figure 1:**
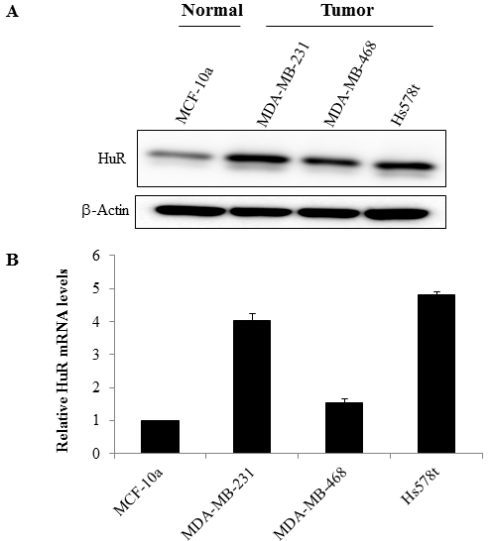
HuR is overexpressed in TNBC cells **A.** Analysis of whole cell extracts for HuR by western blotting showed HuR expression was higher in TNBC cell lines compared to normal mammary epithelial cells. Actin was used as loading control. **B.** RNA was extracted from breast cancer cell lines and relative expression of HuR mRNA was assessed by quantitative RT-PCR analysis (normalized against GAPDH). Data are expressed as means ± SE of minimum three independent experiments. Asterisk denotes significance (*p* ≤ 0.05).

HuR expression in human in TNBC cell lines (MDA-MB-231, MDA-MB-468 and Hs578t) and the normal mammary epithelial cell line (MCF-10a) was evaluated both by Western blot and by real-time quantitative (q) PCR. Total HuR protein levels were higher in the TNBC cell lines compared to the normal mammary epithelial cell line (Figure 1A). HuR mRNA levels were also markedly higher in the TNBC cell lines compared to the normal cell line (Figure 1B). Among the tumor cell lines examined, HuR mRNA levels were higher in MDA-MB-231 and Hs578t cells compared to MDA-MB-468 cells.

### Silencing HuR enhances radiosensitivity of TNBC cells *in vitro*

Given the elevated levels of HuR in TNBC cell lines, we investigated the effect of HuR knockdown on the growth of TNBC cells. Prior to conducting siRNA studies, we tested the specificity and the effectiveness of the siRNA against HuR by using three different HuR siRNAs (siHuR) and compared to scrambled siRNA (siScr) in MDA-MB-231 cells. As shown in Supplementary Figure S1, all three HuR siRNAs effectively reduced HuR protein expression compared to siScr. However, the inhibitory activity of all three HuR siRNAs was comparable and based on the results obtained we chose to use siRNA # 1 in all of the studies described herein.

**Figure 2:**
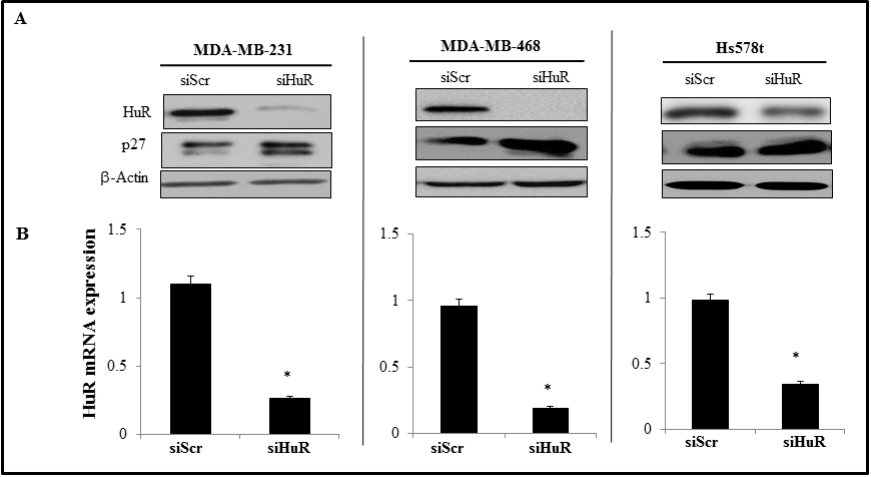
Effect of HuR silencing on the expression of HuR protein and mRNA **A.** siHuR- treated TNBC cells showed reduced HuR protein expression with concomitant increase in p27 expression compared to siScr-treated cells. Actin was used as a loading control. **B.** HuR mRNA was significantly downregulated in siHuR-treated TNBC cell lines compared to siScr-treated cells. Asterisk denotes significance (*p* ≤ 0.05).

SiRNA-mediated silencing of HuR in TNBC cell lines (MDA-MB-231, MDA-MB-468 and Hs578t) resulted in significant reduction in HuR mRNA and protein expression levels compared to scrambled siRNA (siScr)-treated cells (Figure 2A, 2B; *p* < 0.05). Correlating with HuR suppression in the three tumor cell lines was a marked increase in p27 protein expression, a molecular downstream target that is regulated by HuR (Figure 2A).

**Figure 3:**
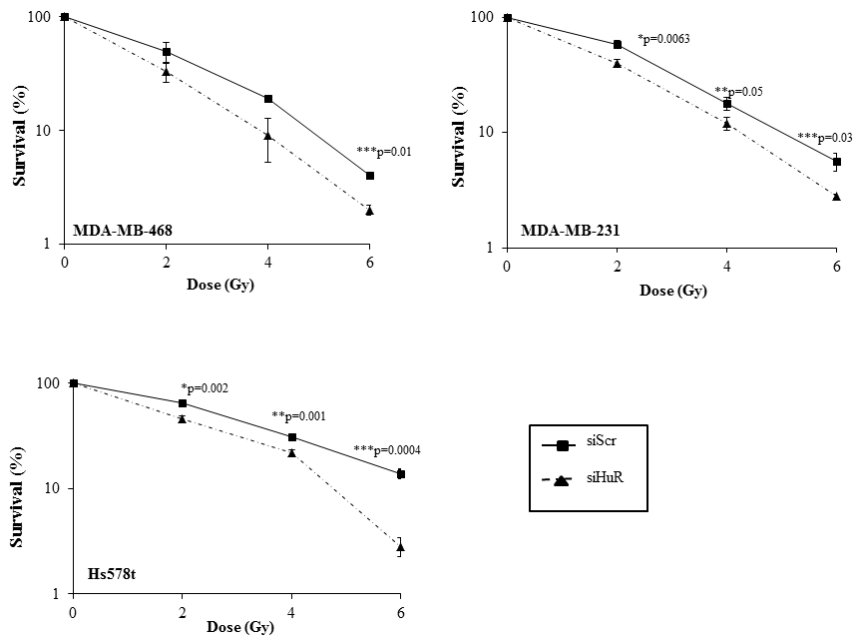
HuR silencing radiosensitizes human triple negative breast cancer cells MDA-MB-468, MDA-MB-231 and Hs578t cells transfected with siHuR showed significant radiosensitization compared to siScr-transfected cells. Data represent the average of three independent experiments each plated in triplicate: solid line, siScr; dotted line, siHuR. Error bars represent ±SE (*p ≤ 0.05).

We next investigated the outcome of HuR silencing on the radiosensitivity of TNBC cells by assessing their clonogenic survival potential. Knockdown of HuR significantly suppressed the clonogenic survival of all three TNBC cell lines compared to survival in siScr-treated cells (Figure 3). Growth suppression was observed at all of the radiation doses tested in the three cell lines albeit to varying degree. In MDA-MB-231 cells, the survival factor (SF) at 2 Gy was reduced from 59 ± 4% in the siScr-treated cells to 40 ± 3% (*p* < 0.05) in the siHuR-treated cells (Figure 3). In MDA-MB-468 cells, the SF2 was reduced from 49 ± 10% in the siScr-treated cells to 33 ± 7% in siHuR-treated cells (*p* < 0.05) while in Hs578t cells, the SF2 values were reduced from 65 ± 2% in siScr-treated cells to 46 ± 3% (*p* < 0.05) in siHuR-treated cells (Figure 3). The survival enhancement ratios were calculated at 10% cell survival by dividing radiation dose of the siScr plus radiation survival curve with that of the corresponding siHuR plus radiation curve. The survival enhancement ratio was 1.22 for MDA-MB-231 cells, 1.2 for MDA-MB-468 and 1.38 for Hs578t cells respectively.

To further confirm siHuR knockdown contributes to radiosensitization, we conducted HuR rescue studies. Exogenous overexpression of wild-type HuR in MDA-MB-231 cells using a plasmid expression vector (HuR-TAP) followed by radiation demonstrated a tendency for increased radioresistance (Supplementary Figure S2) when compared to control cells that were transfected with control plasmid DNA (Empty-TAP). These results show that silencing of HuR radiosensitized the cancer cells.

### HuR silencing modulates downstream targets of HuR

**Figure 4:**
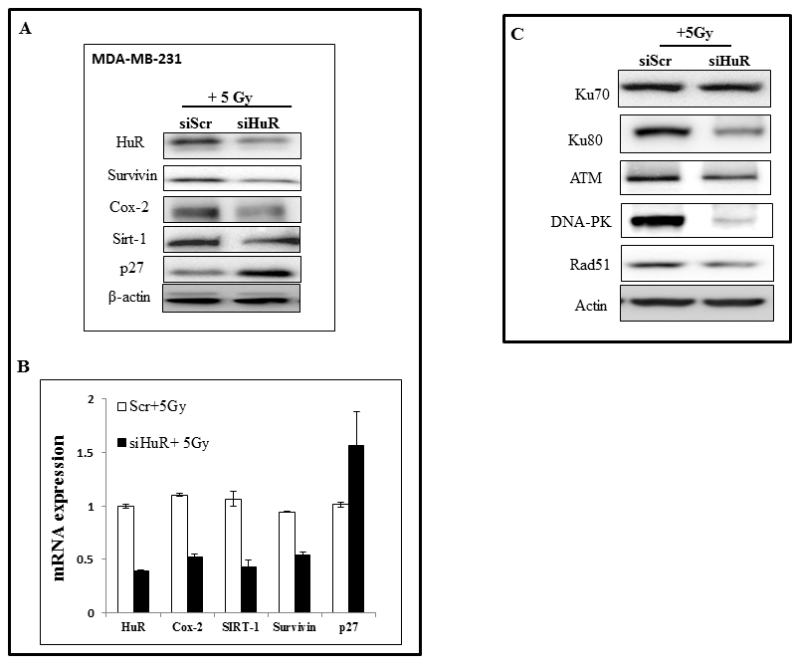
Modulation of HuR targets and DNA repair proteins upon siHuR and radiation treatment MDA-MB-231 cells transfected with siScr or siHuR were irradiated and harvested 2 hours later. **A.** Western blot analysis showing suppression of HuR target proteins. **B.** RT-qPCR analysis of HuR and HuR-target mRNAs. **C.** Suppression of DNA repair proteins after siHuR plus radiation treatment compared to siScr plus radiation treatment.

We next determined the effects of HuR silencing when combined with radiation (5 Gy) on the expression levels of HuR-regulated molecular targets (survivin, COX-2, Sirt-1, and p27) by western blot and qRT-PCR analyses in MDA-MB-231 cells. In siHuR plus radiation-treated cells, a marked reduction in survivin, COX-2 and Sirt-1 was observed both at the mRNA and protein level when compared to siScr plus radiation treated cells (Figure 4A, 4B). In contrast, expression of the CDK inhibitor p27 was observed to be increased in siHuR plus radiation-treated cells compared to siScr plus radiation treated cells. The observed increase in p27 expression on HuR inhibition is in keeping with HuR-mediated repression of p27 translation [46]. These results show HuR silencing affected the expression of its downstream targets.

### Modulation of DNA repair gene expression by siHuR may influence TNBC radiosensitivity

Studies from our laboratory and others have previously demonstrated a role for DNA repair proteins in radioresistance and that suppression of these proteins enhances the radiosensitivity in human tumor cells [47-49]. Based on these reports and our present observation that HuR silencing caused radiosensitization of TNBC cells, we examined the effect of siHuR plus radiation treatment on the expression of proteins (Ku70, Ku80, ATM, DNA-PK and Rad51) known to be involved in the repair of radiation-induced DSBs. Western blot analysis showed a marked reduction in the expression of Ku80, ATM, DNA-PK and Rad51 proteins in siHuR plus radiation-treated MDA-MB-231 cells compared to siScr plus radiation-treated cells (Figure 4C).

No appreciable change in Ku70 protein expression level was observed between siHuR and siScr-treated cells. These results demonstrate siHuR when combined with radiation reduces DNA repair protein expression levels and likely contributes to radiosensitization of TNBC cells.

### HuR depletion prolongs the expression of γH2AX foci and enhances radiation-induced DSBs

**Figure 5:**
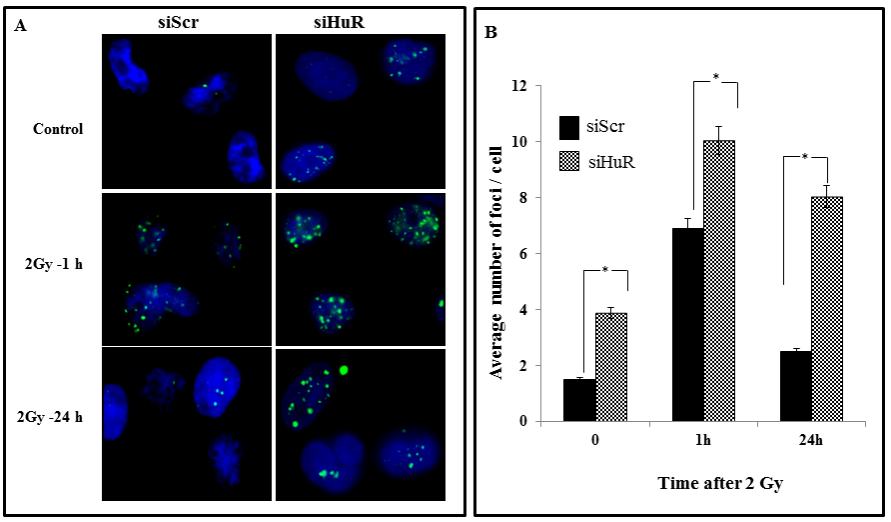
HuR depletion prolongs γ-H2AX expression Sub-confluent MDA-MB-231 cells were transiently transfected with siScr or siHuR (100 nM for 24 h) followed by a single 2 Gy dose of radiation. Samples were then incubated for various times after irradiation and stained for γ-H2AX foci **A.** Representative photomicrographs of MDA-MB-231 from various treatment conditions and time points from three independent experiments are shown. Blue stain: DAPI (nuclei). Green stain: γ-H2AX foci. **B.** γ-H2AX foci were quantified and plotted as the number of foci per nucleus. Mean ± SE number of foci per nucleus is shown. Asterisk denotes significance (*p* ≤ 0.05).

Since HuR knockdown reduced the DNA repair proteins, we hypothesized that HuR-mediated radiosensitization is due to a delay in the repair of the DSBs induced by radiation, suggesting an involvement of the DNA damage and repair pathway ToγH2AX foci, an indicator commonly utilized to assess DNA DSBs inflicted by ionizing radiation, in MDA-MB-231 cells treated with siScr or siHuR with radiation (2 Gy). The cells were immunostained for γ-H2AX at 1h, and 24 h post-radiation. The siScr- and siHuR-treated cells that did not receive radiation treatment served as controls. As shown in the micrographs in Figure 5A, γ-H2AX foci were detected in both siScr- and siHuR-treated cells (control) with number of γ-H2AX foci being greater in siHuR-treated cells (*p* < 0.05). However, when the cells were subjected to radiation, a significant increase in γ-H2AX foci was observed in siHuR-treated cells at both 1 h and 24 h after radiation compared to siScr-treated cells receiving radiation (Figure 5A, 5B; *p* < 0.05). In siScr-treated cells, a marked increase in γ-H2AX foci was observed at 1h after radiation treatment when compared to control cells that however reduced at 24 h after receiving radiation treatment and reached levels comparable to that observed in siScr-treated control cells (Figure 5A, 5B). Our results show that siHuR treatment induces γ-H2AX foci that is further enhanced and sustained over time following radiation treatment compared to siScr treatment.

**Figure 6:**
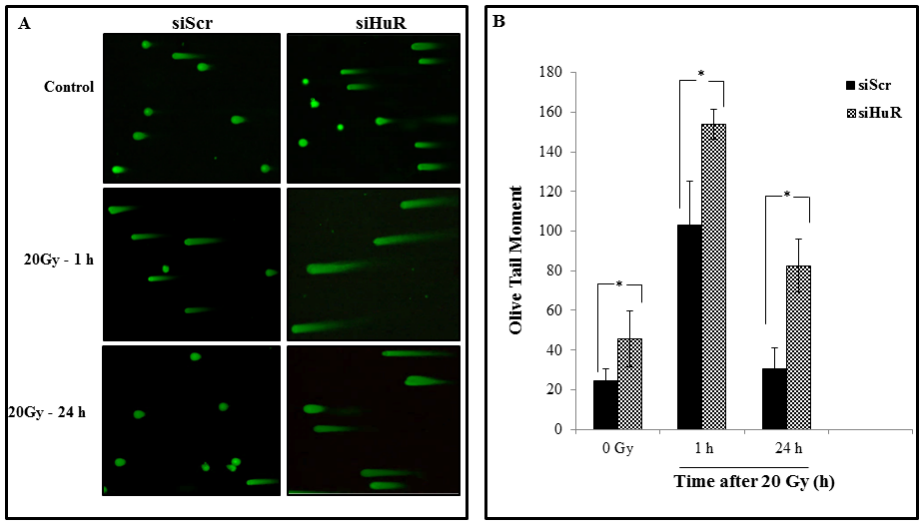
Silencing HuR enhances radiation induced DSBs MDA-MB-231 cells were transfected with either siScr or siHuR and 24 hours later were irradiated at 20 Gy and harvested at the indicated times. **A.** Representative comet images of DSBs detected by neutral comet assay demonstrate the kinetics of tail moment in siScr or siHuR transfected cells at 0, 1 and 24 hours after irradiation. **B.** Distribution of DNA damage in cells treated as described in panel A. Olive tail moment (OTM) values were determined following the algorithm (olive tail moment = tail mean - head mean) tail% DNA/100) using Casplab software. Error bars represent SE. Asterisk denotes significance (*p* ≤ 0.05).

To further examine whether the combination of siHuR and radiation increases DNA damage, we performed neutral comet assay which detects DNA DSBs. Elevated level of DNA damage was observed in siHuR-treated MDA-MB-231 cells compared to the siScr control cells not receiving radiation, which showed largely non-fragmented DNA (Figure 6A, 6B). Combined siHuR plus radiation (20 Gy) however resulted in higher DNA damage, as evidenced by longer comet tails and increase in tail length compared to siScr plus radiation treated cells at 1 h, and 24 h following radiation (Figure 6A, 6B; *p* ≤ 0.05). These results suggest that the repair of radiation-induced DNA damage was significantly suppressed and prolonged upon HuR knockdown in tumor cells. Thus, siRNA-mediated HuR knockdown coupled with radiation treatment involves inhibition of DNA repair resulting in radiosensitization of tumor cells.

### HuR knockdown inhibits intracellular Thioredoxin reductase and enhances radiation-induced ROS production

**Figure 7:**
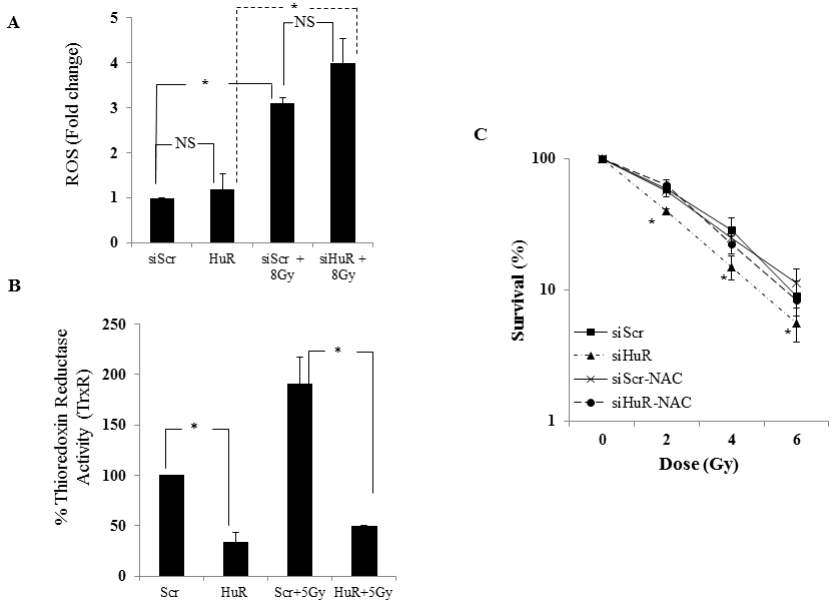
HuR knockdown inhibits thioredoxin reductase and enhances radiation-induced ROS production **A.** MDA-MB-231 cells transfected with siScR or siHuR were incubated with 20 μM of H2DCFDA for 1 hour at 37°C. The cells were then irradiated at 8 Gy, harvested, and fluorescence activity measured at 495 nm excitation and 529 nm emission. Increase in ROS production was observed in siHuR-treated cells compared to siScr. ROS production was significantly increased when combined with radiation in both siScr and siHuR-treated cells. “NS” denotes no significance. **B.** Thioredoxin reductase (TrxR) activity was reduced in siHuR-treated MDA-MB-231 cells both in the presence and absence of radiation compared to siScr-treated cells. Asterisk denotes significance (*p* ≤ 0.05). **C.** siScr- and siHuR-treated cells were treated or treated for 1 hour with 10 μM NAC prior to radiation. The cells were subsequently radiated at 2, 4 and 6 Gy and subjected to clonogenic cell survival assay. siHuR-mediated radiosensitization was significantly abrogated in NAC-pretreated cells. Asterisk denotes significance (*p* ≤ 0.05).

Since siHuR inhibited DNA repair and prolonged expression of γ-H2AX we next examined if the radiosensitization observed in our model system was mediated through modulation of the intracellular oxidative stress response pathway. MDA-MB-231 cells were treated with siScr or siHuR and generation of ROS in the cells with or without radiation was measured 30 minutes post-radiation using the cell-permeant 2′,7′-dichlorodihydrofluorescein diacetate (H_2_DCFDA) dye. MDA-MB-231 cells treated with siScr or siHuR plus radiation displayed a significant increase in ROS compared with siScr or siHuR-treated cells that did not receive radiation treatment (Figure 7A; siScr *vs*. siScr/Gy = *p* ≤ 0.003; siHuR *vs*. siHuR/Gy *p* ≤ 0.007). However, ROS production did not significantly change in HuR silenced cells relative to siScr cells (Figure 7A, siScr *vs*. siHuR = *p* ≤ 0.19). Furthermore, upon radiation no significant change in ROS production was observed between siHuR and siScr cells (Scr/Gy *vs*. HuR/Gy = *p* ≤ 0.23). As shown in Figure 7A, though siHuR knockdown enhanced the generation of radiation induced ROS compared to the siScr cells these changes did not reach significance.

Since ROS plays a role in the cellular damage induced by radiation, factors that regulate ROS may be important for the protection of cells against radiation-induced damage [5, 50]. We next tested if siHuR affected the intracellular redox balance through alteration of cellular thiols. Thioredoxin reductase (TrxR) is one such enzyme that reduces and activates thioredoxin which binds to ROS and thus protects cells against oxidative stress [11, 12]. As seen in Figure 7B, HuR silencing in MDA-MB-231 cells significantly reduced TrxR activity compared to the siScr-treated cells (siScr *vs*. siHuR = *p* ≤0.018). Further, the radiation induced increase in TrxR activity observed in siScr-treated cells was significantly suppressed in siHuR-treated cells (Figure 7B; siScr/Gy *vs* siHuR/Gy = *p* ≤ 0.034). These results demonstrate that HuR silencing not only inhibits TrxR in tumor cells but maintains its suppressive activity on radiation induced TrxR activity thereby producing radiosensitization effects on TNBC cells.

### Pretreatment with N-Acetyl Cysteine abrogates siHuR-mediated radiosensitization

To determine the contribution of ROS to siHuR-mediated radiosensitization, siHuR- and siScr- treated MDA-MB-231 cells were either not treated or treated with the ROS scavenger N-Acetyl Cysteine (NAC, 10 mM) for 1 h before subjecting the cells to radiation. NAC pretreatment greatly abrogated the radiosensitizing effects of siHuR when compared to siHuR-treated cells not receiving NAC (Figure 7C; *p* < 0.05). No significant change was observed in siScr-treated cells that received NAC compared to those that did not receive NAC treatment. These results clearly demonstrate that the enhanced radiosensitivity observed after silencing HuR in TNBC cells involved ROS generation and DNA damage.

## DISCUSSION

HuR/ELAV1, a ubiquitously expressed RNA-binding protein that regulates the expression of genes involved in key cellular processes, has been implicated in regulating resistance to chemotherapeutic agents in various cancers, but whether HuR modulates radiosensitivity of cancer cells is not known. In this study we investigated whether HuR suppression could enhance the radiosensitivity of TNBC cells *in vitro*. We first assessed HuR expression in TNBCs and compared them to the normal mammary epithelial cells (Figure 1). Distinct overexpression of HuR was seen in the TNBC cell lines. We further tested the effects of silencing HuR on radiosensitization of TNBC cells and observed that all three TNBC lines were radiosensitized upon HuR knockdown (Figure 3). Given the pleiotropic effects of HuR on various cellular processes, its down-regulation could have a multitude of effects and accordingly a broad range of HuR targets may help confer resistance to TNBC cells exposed to ionizing radiation. We, therefore, examined the effect of silencing HuR on several mRNAs encoding proteins that play a role in cell proliferation, apoptosis and DNA damage repair in breast cancer cells. Silencing HuR significantly decreased expression of its mRNA targets including COX-2, survivin, and SIRT-1 with a further decrease after radiation strengthening the idea that HuR influences cell survival in this paradigm (Figure 4). Sirtuin-1 (SIRT1), is an nicotinamide adenine dinucleotide (NAD+)-dependent deacetylase that is overexpressed in a variety of cancers including breast cancer and plays an important role in DNA damage response and genome integrity by maintaining proper chromatin structure and DNA damage repair foci formation [51, 52]. Studies have also shown that Sirt1 knockout mice die in early postnatal stages and that SIRT1-defective or knockdown cells are more sensitive to several DNA damaging agents [53, 54]. We observed a decrease in SIRT1 upon HuR knockdown, in the presence and absence of radiation, compared to the Scr controls. This decrease in SIRT-1 expression can lead to an impaired ability to repair DNA DSBs thereby contributing to the observed radiosensitization upon HuR knockdown. We also observed an accumulation of p27 upon HuR silencing, in the presence and absence of radiation, similar to what has been reported in the literature [34].

Growing evidence suggests that ionizing radiation kills cancer cells by directly causing DNA DSBs or by producing ROS which cause DNA damage indirectly [5, 50, 55]. Radiation-induced DNA DSBs are considered to be the most crucial cellular damage which induce cell killing and therefore cellular radioresistance is often correlated with efficiency to repair DNA DSBs [56, 57]. These DSBs are primarily repaired by two key pathways: non-homologous end joining (NHEJ) or homologous recombination (HR) pathways. Essential components of NHEJ are Ku70, Ku80 and DNA-PK and loss or mutations in any of the three subunits can lead to extreme radiosensitivity and DSB repair deficiency [58, 59, 60]. We observed a suppression in the levels of Ku80 but not Ku70 upon HuR silencing with and without radiation in MDA-MB-231 cells. However, a reduction in DNA-PK expression was observed only in the siHuR plus radiation group, suggesting that inhibition of the NHEJ pathway by siHuR augments tumor radiosensitivity. Assessment of the levels of Rad51 and ATM (key proteins in the HR pathway), also demonstrated a suppression in their levels upon combination with radiation. The data indicates that inhibition of the NHEJ and HR pathway by siHuR could be a possible mechanism underlying the observed radiosensitization.

We also evaluated the involvement of DNA repair in siHuR-mediated radiosensitization using γ-H2AX as a molecular biomarker. H2AX is a variant form of the nucleosomal protein, histone H2A, which upon phosphorylation on its S139 site (γH2AX) by ataxia telangiectasia mutated (ATM) in response to DNA DSBs can be detected as ‘γ-H2AX foci’[62]. γ-H2AX has been established as a highly sensitive indicator to monitor the induction and repair of DNA DSBs induced by low doses of radiation. Constitutive expression of histone γ-H2AX has been shown to indicate disruption of the DNA damage repair pathway and/or genetic instability [63]. Since we observed a reduction in the expression of DNA repair proteins upon knockdown of HuR, we evaluated the delay in the repair of DSBs upon radiation by examining the γ-H2AX foci expression in MDA-MB-231 cells. siHuR treatment increased the expression of radiation-induced γH2AX foci at all times post radiation and sustained γH2AX foci were observed even at 24 h (Figure 5). This persistence of foci in HuR silenced cells suggests an inhibition of the DSB repair pathway which correlates with enhanced radiosensitivity. To confirm that these foci measurements depict inhibition of DSB repair, we used neutral comet assay which under neutral pH conditions detects DNA fragments that occur due to DSBs [64]. Consistently larger and longer tails were observed in HuR- silenced cells compared with control cells alone or in combination with radiation (Figure 6), indicating that HuR radiosensitizes TNBC cells by suppressing the cellular capacity for repairing radiation induced DSBs.

It is well documented that ionizing radiation (IR) causes an increase in intracellular reactive oxygen species (ROS) production resulting in oxidative damage to lipids, proteins and DNA subsequently leading to DNA DSBs [5,50]. In this report we show that silencing HuR leads to elevated levels of ROS in MDA-MB-231 cells with further increase when combined with radiation (Figure 7). ROS plays an important role in the cytotoxic action of IR and the potential adverse effects of ROS are prevented by different cellular antioxidant systems like glutathione, thioredoxin reductase, glutathione peroxidase, catalase, and superoxide dismutase, which form the first line of defense against ROS induced oxidative stress in cells [8, 55, 65]. These antioxidants help cells in scavenging ROS and salvaging biomolecules from oxidative damage. One of the most versatile protectors of such antioxidants is Thioredoxin Reductase (TrxR), which plays a key role in maintaining redox-regulated cellular functions, including transcription, DNA damage recognition and repair, proliferation, and apoptosis [11, 12, 66]. TrxR is often upregulated in human cancers and preclinical studies have shown that the inhibition of TrxR activity can increase the radiosensitivity of tumors cells while elevated intracellular Trx levels confer resistance to radiation [12-14, 67]. Additionally, agents that selectively target TrxR have shown promising results as anticancer drugs in preclinical and clinical studies when used alone or in combination with IR [67-69]. Rockwell et al [69] showed that motexafin gadolinium, a potent inhibitor of TrxR, enhances tumor cell response to radiation and is currently being tested in phase I clinical trials for patients with brain metastases from lung and breast cancer. Smart et al., [14] showed that HeLa cells overexpressing the wild-type form of TrxR but not the dominant-negative form were more resistant to the lethal effects of radiation. They also saw a significant increase in the radiosensitivity of HeLa and FaDu cells upon knockdown of TrxR, further supporting the role of TrxR as a major determinant of intrinsic tumor cell radiosensitivity. Therefore, we examined TrxR function in MDA-MB-231 cells with and without radiation and found that silencing HuR alone reduced TrxR activity and that HuR silencing in combination with radiation cooperatively inhibited TrxR activity. These results are in agreement with previous findings where knockdown of TrxR levels caused a significant increase in radiosensitivity of tumor cells [70]. After silencing HuR, depleted TrxR levels in the MDA-MB-231cells (Figure 7) would be expected to contribute to the accumulation of ROS. As confirmation of this mechanism, pretreatment with NAC, a scavenger of free radicals [61], diminished siHuR-induced radiosensitization (Figure 7). Loss of ROS generation was also observed providing further evidence that ROS levels are important in mediating the radiation response observed with HuR knockdown. At the linear energy transfer level of essentially all clinically used photon irradiation and that used in these studies, the indirect mechanism will greatly predominate.

In conclusion we demonstrate that silencing HuR in TNBC cells elicits oxidative stress and DNA damage resulting in radiosensitization.

## MATERIALS AND METHODS

### Cell lines

Human breast cancer cell lines, MCF-10a, MDA-MB-231, MDA-MB-468 and Hs578t were purchased from the American Type Culture Collection (Manassas, VA) and grown in alpha-Minimum Essential Media containing 10 % fetal bovine serum, 2 mmol/L L-Glutamine and 2 mmol/L Penicillin-Streptomycin. Cultures were maintained at 37°C in an atmosphere of 5 % CO_2_ and 95 % room air.

### siRNA transfection

The transfection of MDA-MB-231, MDA-MB-468 and Hs578t cells with human ELAV1siRNA (siHuR) and non-targeting siRNA#3 (siScr) (GE Dharmacon, Lafayette, CO) was performed in six well plates using DharmaFECT 2 transfection reagent (GE Dharmacon, Lafayette, CO) according to manufacturer's instructions. Cells were transfected with siRNA (100 nM) in serum free medium. Six hours after transfection, the media was replaced with fresh medium containing 2 % serum. The next day the cells were irradiated (5 Gy) and harvested for further experiments.

### Clonogenic survival

The effectiveness of the combination of siHuR and ionizing radiation was assessed by clonogenic assays as described previously [72]. Briefly, cells were treated with siScr or with siHuR at 100 nM for 24 h and then irradiated with a high dose-rate ^137^Cs unit at room temperature. Cells were then trypsinized and counted and known numbers were seeded onto 60-mm dishes in triplicate. The dishes were then returned to the incubator and allowed to grow macroscopic colonies undisturbed for 14 days. Colonies were fixed and stained with 0.5 % crystal violet in methanol. The number of colonies formed in each treatment group was counted, with a cutoff of 50 viable cells per colony. The plating efficiency and survival of cells irradiated at doses of 0, 2, 4 and 6 Gy were calculated as percentages. The percentage plating efficiency (PE) and the fraction surviving a given treatment were calculated based on the survival of non-irradiated cells treated with siScr or siHuR. Survival curves were generated after normalizing for the cytotoxicity generated by siScr alone. Data presented are the mean ± SE from a minimum of three independent experiments, each done in triplicate.

### Western blot analysis

Cells were harvested after treatment with siScr or siHuR alone (100 nM), or in combination with radiation, rinsed in ice-cold PBS and lysed in RIPA buffer containing 25 mM Tris HCl pH 7.6, 150 mM NaCl, 1 % NP-40, 1 % sodium deoxycholate, 0.1 % SDS with Halt Protease Inhibitor Cocktail and Halt Phosphatase Inhibitor Cocktail (Pierce, Thermo Scientific, IL). The lysates were centrifuged at 14,000 rpm to remove any cellular debris. Protein concentrations of the lysates were determined by the BCA protein assay kit (Thermo Scientific, IL). Equal amounts of protein were separated by 12 % SDS-PAGE, transferred to Immobilon PVDF membranes (Millipore, Bedford, MA). Nonspecific-binding sites on the membrane were blocked in 5 % nonfat dry milk in Tris (20 mmol/L)-buffered saline (150 mmol/L, pH = 7.4) with 0.05 % Tween (TBS with Tween) for 1 h at room temperature. Protein signals were detected by incubating the membrane in primary antibody in 5 % nonfat dry milk overnight at 4°C. Antibodies against HuR, β-actin, p27 and Rad51 (Santa Cruz Biotechnology, Dallas, TX), γ-H2AX (EMD Millipore, Billerica, MA), Survivin, COX-2, SIRT-1, Ku70 and Ku80 and DNA-PK (Cell Signaling Technology, Beverly, MA), and ATM (Gene Tex, Irvine, CA) were purchased and used. After washing, the membrane was incubated for 1 h with the appropriate peroxidase-conjugated secondary antibody (GE Healthcare Life Sciences, PA). The membrane was then developed by enhanced chemiluminescence with ECL plus Western Blotting Detection Reagents (Amersham Pharmacia Biotech, Arlington Heights, IL) on a Syngene G-Box (Syngene, MD).

### Quantitative polymerase chain reaction (QPCR)

Total RNA was isolated from treated cells using TRIzol reagent (Life Technologies, Grand Island, NY) and was subjected to reverse transcription (RT) using Omniscript Reverse Transcription Kit (Qiagen, CA) and the complementary DNA (cDNA) was subsequently used to perform real-time, quantitative (q)-PCR (Bio-Rad CFX96™ TouchReal-Time PCR Detection System) with SYBR™ chemistry using PerfeCTa SYBR Green Fast Mix (Quanta Biosciences, MD). The primers for amplifying human HuR, COX-2, SIRT-1, Survivin, p27 and GAPDH (Table 1) were from IDT Technologies (Coralville, IA). The comparative C_t_method was used to calculate the relative abundance of mRNA compared with that of GAPDH expression. The experiment was performed in triplicate and changes in mRNA were expressed as fold change relative to control ± the standard deviation (SD).

**Table 1:** Oligonucleotide primer sequence used in polymerase chain reaction

Gene	Primer sequence
HuR Forward HuR Reverse	5′ ATGAAGACCACATGGCCGAAGACT 3′ 5′ AGTTCACAAAGCCATAGCCCAAGC 3′
COX-2 Forward COX-2 Reverse	5′ CCCTTGGGTGTCAAAGGTAA 3′ 5′ GCCCTCGCTTATGATCTGTC 3′
SIRT-1 Forward SIRT-1 Reverse	5′ TGAGGCACTTCATGGGGTATGG 3′ 5′ TCCTAGGTTGCCCAGCTGATGAA 3′
Survivin Forward Survivin Reverse	5′ TCATAGAGCTGCAGGGTGGATTGT 3′ 5′ AGTAGGGTCCACAGCAGTGTTTGA 3′
p27 Forward p27 Reverse	5′ TGG AGA AGC ACT GCA GAG AC 3′ 5′ GCG TGT CCT CAG AG T TAG CC 3′
GAPDH Forward GAPDH Reverse	5′ AGCCTCAAGATCATCAGCAATGCC 3′ 5′ TGTGGTCATGAGTCCTTCCACGAT 3′

### Immunofluorescent staining for γ-H2AX

γ-H2AX was detected as previously described [47]. Briefly, cells were grown and treated with 100 nM siScr or siHuR for 24 h on coverslips placed in 35 mm dishes. At specified times the cells were irradiated at 2 Gy, media was aspirated, and cells were fixed in 1 % paraformaldehyde for 10 minutes at room temperature. After the paraformaldehyde was aspirated, the cells were fixed in 70 % ethanol for 10 minutes at room temperature followed by treatment with 0.1 % NP40 in PBS for 20 minutes. After two 5-minute rinses in PBS, the cells were incubated in blocking buffer (5 % BSA in PBS) for 1 h. Next, the cells were incubated in γ-H2AX primary antibody (EMD Millipore, MA) at a dilution of 1:300 in 5 % BSA in PBS overnight at 4°C with gentle shaking. Cells were then washed three times in PBS before incubating in the dark with Alexa Fluor −488 labeled secondary antibody at a dilution of 1:300 in 5 % BSA in PBS for 2 h. Nuclei were counterstained with 4′6-diamidino-2-phenylindole dihydrochloride (DAPI, 1 μg/ml) in PBS for 5 minutes, and the coverslips were mounted on slides with Vectashield (Vector Laboratories, CA). Slides were examined on a Nikon fluorescent microscope (Nikon Instruments, NY). Images were captured by a CCD camera and imported into Image J (NIH) analysis software. To quantify γ-H2AX foci, minimum of 50 nuclei per treatment were evaluated.

### Comet assay

Radiation-induced DSBs were detected using a Comet Assay kit (Trevigen, Gaithersburg, MA) according to the manufacturer's instructions. Briefly, cells were transfected with siScr and siHuR (100 nM) and 24 h later they were irradiated at 20 Gy, harvested, washed twice, and resuspended at 1×10^5^ cells/mL in ice-cold 1X PBS. The cells were then combined with low-melting (LM) agarose at a ratio of 1:10 (v/v) and spread on the Comet Slide. The slides were allowed to solidify for 30 minutes in the dark at 4°C and then submerged in precooled, neutral lysis buffer at 4°C overnight. After lysis, slides were washed in 1X TBE buffer [0.89 mol/L Tris, 0.88 mol/L boric acid, 2 mmol/L EDTA (pH 8.3)] for 15 minutes and subjected to electrophoresis at 1.0 V/cm for 45 minutes. The slides were rinsed with distilled water and placed in 70 % ethanol for 5 minutes, following which they were dried at 37°C for 15 minutes. The slides were then stained with SYBR Green for 30 minutes in dark and comet images were obtained with a Nikon fluorescence microscope with an attached CCD camera. Images were taken using NIS-Elements imaging software (Nikon Instruments, NY) and analyzed using Casplab comet assay software. The Olive Tail Moment was determined for 50 cells in each sample [64].

### Measurement of intracellular ROS

siScr or siHuR transfected cells (100 nM for 24 h) were washed with ice-cold 1X HBSS, and incubated with 20 μM 2′,7′-dichlorofluorescein diacetate (DCFDA) dye (Molecular Probes, NY) in fresh HBSS for 60 minutes at 37°C, following which cells were irradiated and ROS levels were determined according to the manufacturer's recommendations. Fluorescence intensity was detected using the Envision fluorescence plate reader (Perkin Elmer, MA) with excitation/emission of 485/530nm. Three independent experiments were performed. Values are shown as mean ± SEM. Statistical significance was determined using two-sided Student's t test, and P<0.05 was considered significant.

### Thioredoxin reductase 1 activity assay

Cells were transfected with siScr or siHuR (100 nM) and 24 h later they were exposed to dose of 5 Gy radiation and thioredoxin reductase activity was measured using the Thioredoxin Reductase Activity Kit (BioVision, CA). Briefly 2×10^6^ cells were homogenized in the kit assay buffer and centrifuged at 10,000g for 15 minutes. The supernatant was combined with the reaction mix and incubated at room temperature for 20 minutes. Optical density was measured at 412 nm at time zero (T0) and time T1 after 20 minutes of incubation and calculations were done according to manufacturer's instructions. Values are shown as mean ± SEM. Statistical significance was determined using two-sided Student's t test, and P<0.05 was considered significant.

### Statistical analysis

Statistical analysis was performed using the t-test (Sigma Plot 5.02v) and described as mean ± standard error of the mean. A difference was regarded as significant if *p* < 0.05.

## SUPPLEMENTARY MATERIAL FIGURES


