# Supplementary material for: HuR silencing elicits oxidative stress and DNA damage and sensitizes human triple-negative breast cancer cells to radiotherapy
Source: Oncotarget. 2016 Aug 30;7(40):64820–35. doi: 10.18632/oncotarget.11706 (PMC5323119; doi:10.18632/oncotarget.11706)
Supplement: Supplementary file 1 [file oncotarget-07-64820-s001.pdf]

HuR silencing elicits oxidative stress and DNA damage and sensitizes human triple-negative breast cancer cells to radiotherapy

Supplementary Material

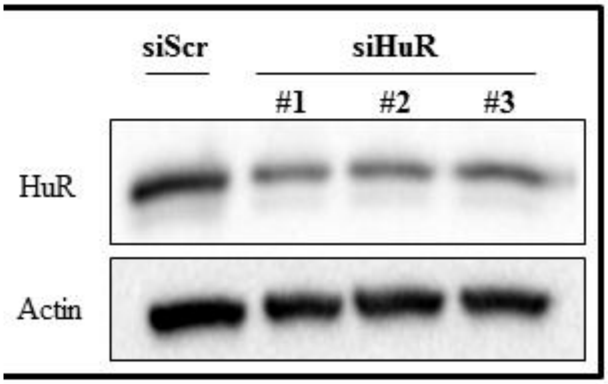

Figure S1: Optimization of HuR siRNA. MDA-MB-231 cells were transfected with three different HuR siRNA (# 1, 2, and 3) and analyzed by western blotting for HuR protein expression at 24 h after treatment. siScr transfected cells served as control. All three HuR specific siRNAs showed equivalent degree of inhibition compared to siScr treated cells. Actin was used as a loading control.

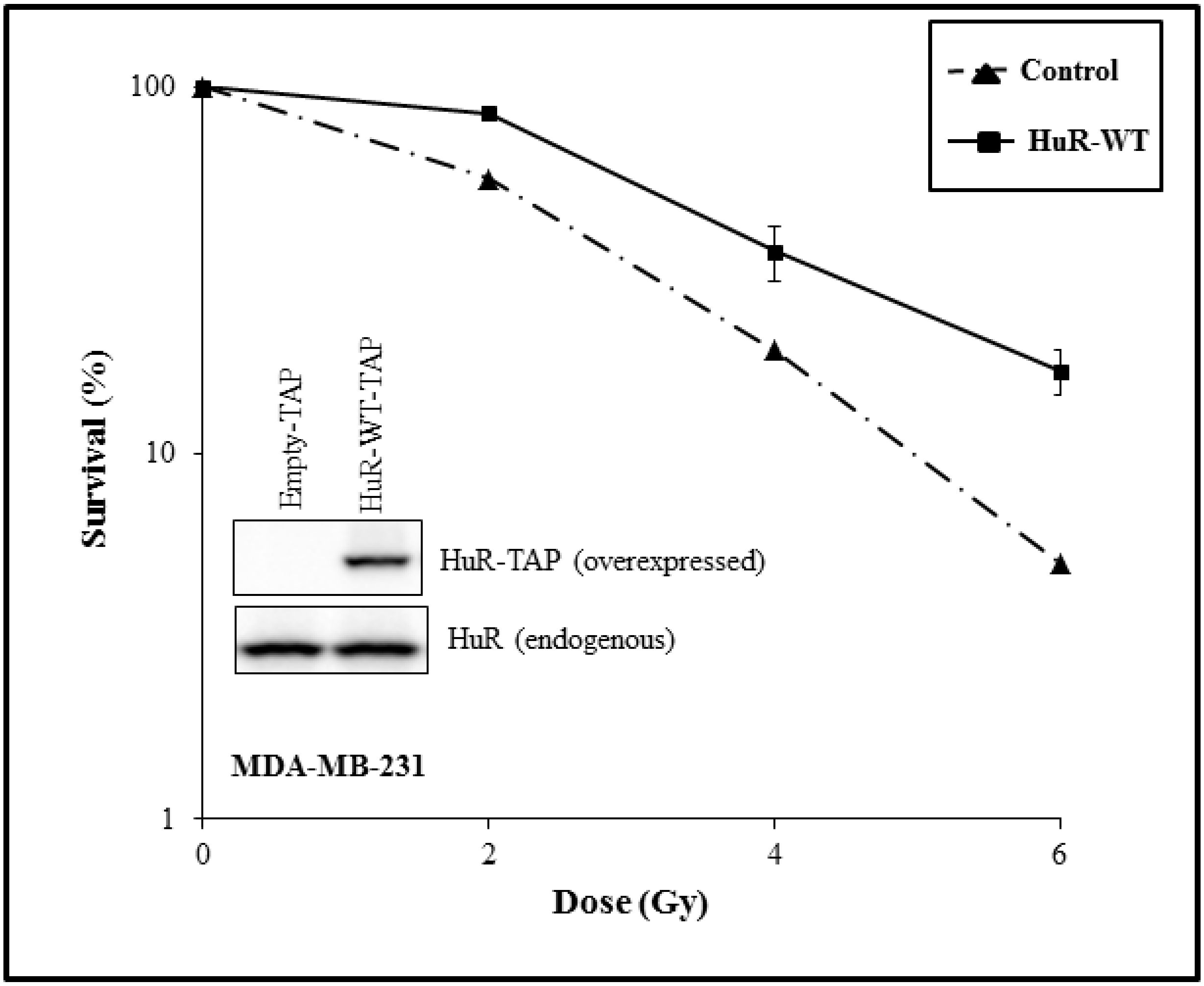

Figure S2: Overexpression of wild-type HuR increases radiation resistance. MDA-MB-231 cells transfected with a wild-type HuR expression vector (HuR-WT-TAP) were exposed to different doses of radiation and compared to cells transfected with empty TAP plasmid (control). Clonogenic survival assay showed a marked increase in radiation resistance in wild-type HuR transfected cells compared to control cells.
